# Effect of foot orthoses on balance among individuals with flatfoot: A systematic review and meta-analysis

**DOI:** 10.1371/journal.pone.0299446

**Published:** 2024-03-08

**Authors:** Chatanun Chinpeerasathian, Phyu Sin Oo, Akkradate Siriphorn, Praneet Pensri

**Affiliations:** Faculty of Allied Health Sciences, Department of Physical Therapy, Chulalongkorn University, Bangkok, Thailand; National Trauma Research Institute, AUSTRALIA

## Abstract

Individuals with flatfoot have impaired proprioception owing to ligament laxity and impaired tendons, which can result in poor balance. Foot orthoses (FOs) have been reported to stimulate plantar mechanical receptors and are used to manage foot overpronation in individuals with flatfoot. However, the results of the use of FOs to improve balance are inconsistent. In this systematic review and meta-analysis, we aimed to identify and investigate the effects of FOs on balance in individuals with flatfoot. Electronic databases were searched for articles published before March 2023. Peer-reviewed journal studies that included adult participants with flexible flatfoot and reported the effects of FOs on balance were included and classified based on the study design: randomized control trials (RCT) and non-RCTs. Four RCT studies were retained, and their methodological quality was assessed (mean, 63.2%; range 47.3%–73.1%: high), as were three non-RCT studies (mean, 54.1%; range, 42.1%–68.4%: high). Meta-analysis was performed by calculating the effect size using the standardized mean differences between the control and FO conditions. Transverse-arch insoles immediately improved static balance after use. However, no immediate significant effect was found for medial archsupport FOs, cuboid-posting FOs, or University of California Berkeley Laboratory FOs during the study period (2–5 weeks) when compared with the controls. The transverse-arch insole is the most effective FO feature for improving static balance. However, the high heterogeneity between study protocols contributes to the lack of evidence for the effects of FO on balance in people with flatfoot.

## Introduction

Pes planus, commonly referred to as flatfoot, is characterized by collapse of the medial longitudinal arch (MLA) of the foot, leading to overpronation of the subtalar joint, rearfoot eversion, and dorsiflexion with forefoot abduction [1,2]. Abnormal foot posture is associated with various physical alterations, including spring ligament laxity, plantar fascia lengthening, gastrocnemius and peroneal muscular stiffness, and posterior tibialis weakness [3]. This condition can induce changes in the movement of the proximal joints, including the ankle, knee, and hip, potentially damaging adjacent tissues such as ligaments and tendons [3,4]. Because of injured ligaments or tendons, individuals with flatfoot exhibit impaired proprioceptive awareness, including postural control, which can result in balance issues [5–7].

Foot orthoses (FOs) have emerged as a promising intervention to enhance balance and prevent falls in individuals with flatfoot by stimulating plantar mechanical receptors, thereby augmenting somatosensory input [8]. The FO aims to optimize the natural alignment of the foot anatomy and its functional correlates, shielding the MLA from abnormal stresses and fostering optimal foot function and stability. Furthermore, FOs can potentially influence foot pronation and lower limb alignment through an interconnected mechanism involving the subtalar joint and tibia, enhancing the orientation and function of the arch [5].

Research has demonstrated a tendency for the center of body mass to shift internally in individuals with a pronated foot, which is a consequence of a fallen MLA. This necessitates adaptations in the MLA structure and function to mitigate the risk of lower-extremity and balance issues [9]. Although the short-term use of FOs has shown immediate improvements in balance [10–12], long-term results are mixed, with some studies reporting benefits after approximately 4 weeks of use [5,14], while others found no significant effects, especially when compared to other interventions [5,13–15]. Despite the potential of FOs to enhance kinesthetic awareness and improve balance, the efficacy of this intervention remains a topic of ongoing debate [9,16,17].

Although previous systematic reviews have explored the effects of FOs on aspects such as pain and functional ability, a gap is noticeable in the literature regarding their impact on balance in adults with flatfoot [18–20]. Addressing this gap is crucial to devise more effective physical therapeutic strategies for this population. Consequently, in this systematic review and meta-analysis, we aimed to evaluate the influence of FOs on balance in adults with flatfoot, to foster a deeper understanding and facilitate more informed clinical treatment decisions.

## Methods

### Information sources and search strategy

This systematic review and meta-analysis was registered with PROSPERO (ID: CRD42023406402) before initiation and adhered to PRISMA standards [21]. From February 1 to April 30, 2023, two reviewers searched the PubMed, EMBASE, Scopus, and Cochrane CENTRAL databases using a strategy that incorporated all MeSH terms and keywords related to “foot orthosis,” “balance,” and “pronated foot” or “flatfoot.” Keywords were combined using the Boolean operators "AND" and "OR" to provide an extensive list of terms for each category (see Table 1 for a PubMed search example).

**Table 1:** Search strategy.

	Concept 1	Concept 2	Concept 3
		AND	AND
Title/abstract	Flatfoot* OR pronated foot* OR pes planus* OR low foot arch*	Balance* OR postural ability* OR stability* OR center of pressure*	Foot orthoses* OR posting* OR insole* OR wedge* OR cushion*

### Eligibility criteria

All identified studies were imported into EndNote 20 (Thomson Reuters, New York, NY, USA). The inclusion criteria were peer-reviewed studies involving adult participants (aged 16–60 years) with pronated or flat feet, examining the effects of FO interventions on balance compared with a control condition (barefoot, shoes only, sham FOs, or other conservative treatments). Studies not in English, those involving participants with neurological, systemic, or degenerative illnesses, finite-element method studies, conference proceedings, review articles, pilot studies, and case studies, were excluded (see Table 2 for details).

**Table 2:** Inclusion and exclusion criteria.

Inclusion	Exclusion
English-language articles Scientific articles published in peer-reviewed journals Experimental study (randomized control trial [RCT] or non-RCT) All kinds of posting foot orthoses or shoe insoles Adult patients with flatfoot (age 16–60 years) Outcomes measured with any kind of tool for balance	Articles in other languages Unpublished articles Case reports Systematic reviews Other interventions Children or older adults Outcomes not measured with any tool

### Study selection, quality and bias study assessment

Two reviewers, C.C. and P.S.O., independently assessed all titles and abstracts based on predetermined inclusion and exclusion criteria. In cases where the title and abstract provided inadequate information, the entire document was evaluated. Any disagreements were addressed by discussion to establish consensus. The methodological quality was assessed using a checklist developed by Downs and Black checklist for randomized and non-randomized trials [22]. This study used modified 18 of 27 items: eight for reporting (items 1, 2, 3, 4, 5, 6, 7, and 10), two for external validity (items 11 and 12), three for internal validity (bias; items 17, 18, and 20), four for internal validity (confounding; items 21, 22, 23, and 24), and one for power (item 27). Items 8, 9, 13, 14, 15, 16, 19, 25, and 26 were removed from the original version because these relate observational studies [23,24]. Each item was assessed as 0 (“no”), 1 (“yes”), or UD (“unable to determine”), with the exception of item 5 for the principal confounders, which was scored 0 (“no”), 1 (“partially”), or 2 (“yes”). The use of a controlled shoe with FOs and testing environment control were identified as the primary confounders in this analysis because these have been shown to affect balance outcomes. Item 27 was scored as 0 or 1. Studies received a score of 1 if a prior power analysis was performed on the sample size. The total quality score for each study was calculated using a maximum of 19 points. Kappa (κ) values were used to determine inter-rater agreement in quality assessments. The level of agreement was evaluated as slight (0.00–0.20), fair (0.21–0.40), moderate (0.41–0.60), substantial (0.61–0.80), or almost perfect (0.81–1.00) [25]. Discrepancies in scores were resolved through discussion until a consensus was reached. Studies with scores of ≥50% were designated as having high methodological quality [24,26]. For the risk of bias assessment, the risk of bias tool within the Cochrane Review Manager (Version 5.4; Copenhagen: The Nordic Cochrane Centre, The Cochrane Collaboration, 2014) was used. Selection bias (random sequence generation and allocation concealment), performance bias (blinding of participants and personnel), detection bias (blinding of outcome assessment), attrition bias (incomplete outcome data), reporting bias (selective reporting), and other biases (ideally prespecified) were all included in the risk-of-bias tool. To analyze each item, the risk of bias ratings were to identify three outcomes: low risk of bias, uncertain risk of bias, and high risk of bias [27].

### Data collection and analysis

One reviewer (C.C.) extracted and summarized the characteristics and relevant data, including balance outcome values, from the eligible studies and presented them in summary tables. The authors were contacted for missing data and data were extracted from graphs when the results were not explicitly provided. The data were subdivided into subgroups according to the orthosis design and methodological quality. The Cochrane Review Manager was used to analyze quantitative data, producing standardized mean differences and 95% confidence intervals (CIs) for FOs in comparison to control conditions. The effect size (ES) was calculated in a random effects model with standardized mean differences for meta-analysis. Pool ESs were identified as a trivial difference (0–0.2), a small difference (0.2–0.49), a medium difference (0.5–0.79), and a large difference (≥ 0.8) [28]. The meta-analysis demonstrated significant differences when the 95% CI did not cross 0 (P < 0.05). The I^2^ index was used to examine statistical heterogeneity between studies. The I^2^ index of 25–50%, 50–75%, and > 75% indicated low, medium, and high heterogeneity, respectively [29].

## Results

### Study selection

**Fig 1:**
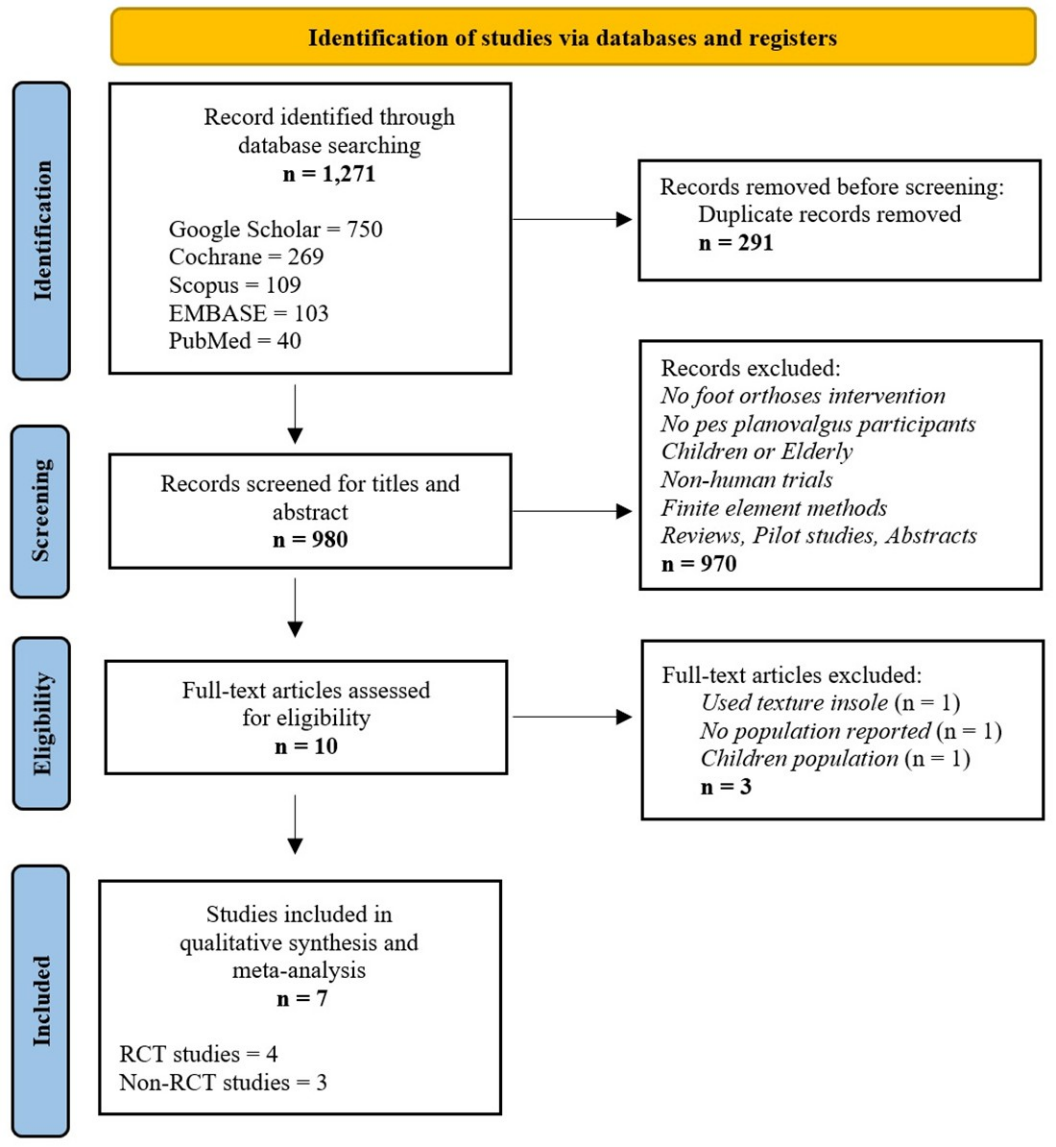
PRISMA flow chart.

**Fig 2:**
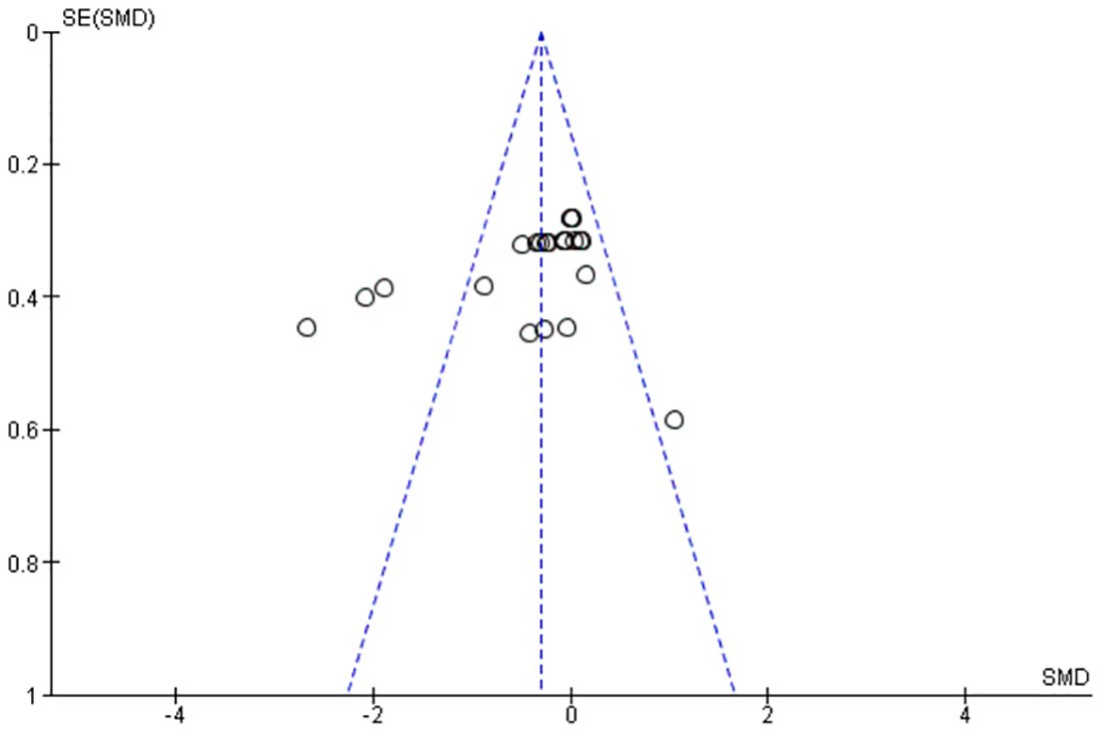
Funnel plot.

The initial search yielded 1,271 papers. After removing 291 duplicates and screening 969 articles by title and abstract, 10 papers remained for full-text review. Following full-text review, three papers were excluded for various reasons. One was excluded because it did not use a posting insole, one was excluded because it did not report the sample size, or one was excluded because it examined the pediatric population. Thus, seven studies (four RCTs and three non-RCTs) were included (Fig 1). These studies were further categorized based on their methodological quality and the geometrical design of the FOs used. The characteristics of the included studies are summarized in Tables 3 and 4. The funnel plot revealed an asymmetry in the distribution of studies, with more studies showing negative than positive effects (Fig 2).

**Table 3:** Summary of included RCT studies.

Author (year)	Sample size (M/F); age mean (SD); foot type	Foot posture eligible	Intervention/control	Setting	Outcomes	Main results
Akbari et al. (2007) [14]	10 females for each group, age group1: 21.6 (2.63), group 2: 22.0 (3.43), flexible flat foot, 2-week intervention	Arch ratio ≤ 0.275	• Rigid medial arch–support FO: ethyl vinyl acetate (EVA) foam with density of 0.2626 g/cm^3^ • Soft medial arch support FO: EVA foam with density of 0.0746 gr/cm3	Two groups: • Group 1: rigid medial-posting FO • Group 2: soft medial-posting FO	• Stability index via Biodex in single-leg stance on unstable surface with bare feet, shoes, and shoes with FOs	• Significantly improved stability while wearing orthoses comparing before and after using FOs. • No significant difference between groups in any conditions was found.
Kim et al. (2016) [15]	7 for each group (10/4), 24.0 (1.9), flexible flat foot, 5-week intervention	Navicular drop test ≥ 10 mm	• Medial arch–support structures. A thermoplastic material. Thereafter, the Aquaplast-T^®^ was cut out using scissors, wetted with 100°C water, and attached to the sole of the participant to copy the foot and the height of the arch. Thereafter, the MLA was made with a shore value of 20° and a height of at least 15 mm (16).	Two groups: • Short foot exercises • Medial arch support	• Navicular drop test • Y-balance test	• Significantly improved balance comparing before and after using both FOs and short foot exercises. • No significant difference in balance between groups was found.
Rome et al. (2007) [13]	25 for each group (20/30), 23.8 (2.2), pronated foot, 4-week intervention	Foot posture index (FPI) > 5	The foot orthoses were rigid prefabricated (Talar Made Orthotics, Chesterfield, UK), composed of high-density EVA with a shore value of 700. The rearfoot wedge was composed of low-density EVA with a shore value of 200. The degree of rearfoot and forefoot wedging was standardized at 50° rearfoot and 0° forefoot.	Two groups: • Rigid rearfoot wedge • No insole	• Mean balance: measures the participant’s ability to stand with an even load. The value is represented as a percentage deviation from an even load and has been reported to be the mean of the 300 balance points measured over 30 seconds. • Sway value: in the medial–lateral and anterior–posterior directions. The sway value was defined as the rate of deviation from the participant’s mean balance over the 30-second test period. *Test in bare feet	• Significant reduction with the intervention group in medial–lateral sway after intervention. • No significant differences between groups in any variables.
Shukla et al. (2021) [5]	15 for each two group (21/9), age M = 18, F = 17.8, flexible flat foot, 4-week intervention	N/A	• UCBLs • Medial arch support	Two groups: • UCBLs • Medial arch support	• Berg Balance Scale • Timed Up and Go (TUG) *Test in bare feet	• Significant difference within groups comparing before and after using both FOs. • Posttest between the parameters (Berg Balance and TUG) showed that UCBLs had a high statistical significance from medial arch support in improving balance and functional parameters.

**Table 4:** Summary of included non-RCT studies.

Author (year)	Sample size (M/F); age mean (SD); foot type	Foot posture eligible	Intervention/control	Setting	Outcomes	Main results
Takata et al. (2013) [10]	20 for each group (20/20), age group: 21.1 (2.63), flat foot, experimental (within-subject)	Bony arch index < 0.21	• BMZ insoles, which supported the cuboid • Superfeet insoles (Impact Trading, Yokohama, Japan), which supported the medial longitudinal arch • No insoles	• Two groups: flatfoot and normal foot • Three conditions within subjects: • 1: using BMZ insoles• 2: using Superfeet insoles• 3: no insoles	• Body sway using a Zebris FDM-SX system (Zebris^®^ Medical GmbH, Isny, Germany) was evaluated based on the center of pressure	• Significantly improved stability while wearing Superfeet insoles comparing the BMZ insoles and no insoles.
Payehdar et al. (2014) [11]	20 (7/13), age M = 22.6 (2.99), F = 23.5 (2.9), flexible flat foot, experimental (within-subject)	FPI > 5	• UCBLs • Modified foot orthoses (MFOs): maximum arch supination stabilization position	Three conditions within subjects (random order): • Shoes only • Shoes with UCBLs • Shoes with MFOs	• Stability index via Biodex system: Total, medial–lateral, and anterior–posterior sway	• No statistical difference in the medial–lateral or anterior–posterior stability indices between foot orthoses and shoed conditions.
Jung et al. (2022) [12]	20 (15/5), 22.0 (3.5), flat foot, experimental (within-subject)	Navicular drop test > 10 mm	• Transverse arch support (FO): Realine insole sports; Realine co., Ltd., Japan); the transverse arch supporter is composed of plastic and compressed foam, and the size is 260–270 mm for men and 230–240 mm for women	Two conditions: • Transverse arch support • Barefoot	• Two-dimensional video analysis: the movements (knee/ankle) of the lower extremity during one-leg standing were recorded using a Samsung Galaxy S20 Note smartphone (Samsung Electronics, South Korea)	• Significantly decreased vertical and horizontal displacement of the knee and vertical displacement of the ankle during one-leg standing in participants with flatfoot after using FOs.

### Quality and bias assessment

The RCT studies had an average methodological quality score of 63.2% (range, 47.3%–73.7%), whereas the non-RCT studies averaged 54.4% (range 42.1%–68.4%). This indicates a generally high methodological quality across both study designs (Tables 5 and 6). Of the RCTs, three were classified as having high methodological quality [13–15], and one was classified as having low methodological quality [5]. Of the non-RCTs, two were classified as having high methodological quality [11,12] and one was classified as having low methodological quality [10]. The reporting items scored high ratings, except for external validity and confounding variables. No study reported a prospective sample size calculation. Inter-rater agreement (Cohen’s kappa coefficient: κ) was almost perfect (κ = 0.855) for the total score (range κ = 0.757–0.910). The same was found for the non-RCTs, with κ = 0.842 for the total score (range κ = 0.802–0.904).

**Table 5:** Methodological quality assessment scores for RCT studies.

Author (year)	Reporting								External validity		Internal validity—bias			Internal validity—confounding				Power	Score (%)	Quality
	1	2	3	4	5^a^	6	7	10	11	12	17	18	20	21	22	23	24	27^b^		
Rome et al. (2004) [13]	1	1	1	1	1	1	1	1	UD	UD	1	1	1	UD	UD	1	UD	UD	63.2	HQ
Akbari et al. (2007) [14]	1	1	1	1	2	1	1	1	UD	UD	1	1	1	1	UD	1	UD	UD	73.7	HQ
Kim et al. (2016) [15]	1	1	1	1	1	1	1	0	UD	UD	1	1	1	1	1	1	UD	UD	68.4	HQ
Shukla et al. (2021) [5]	1	1	1	0	0	1	1	1	UD	UD	1	1	0	UD	UD	1	UD	UD	47.3	LQ

1 = Yes; 2 = No; UD = Unable to Determine; SD: Standard Deviation; HQ: High Quality (Score ≥ 50%); LQ: Low Quality (Score < 50%).

Q1: Clear aim, Q2: Clarity of reporting outcomes, Q3: Clarity of patients’ characteristics, Q4: Describing interventions, Q5: Explaining principal confounders, Q6: Description of main findings, Q7: Estimation and report of random variability, Q10: Reporting actual probability values, Q11: Asked participants well represent the whole population, Q12: The prepared participants well represent the whole recruited participants, Q17: Same time of follow-up, Q18: Appropriate statistical tests, Q20: Accuracy of outcome measures, Q21: Recruiting cases and controls from same population, Q22: Recruiting cases and controls over the same time interval, Q23: Randomized participants to group, Q24: Concealed the intervention from participants and staff, Q27: Sufficient statistical power.

^a^ The score for this question is 0: No, 1: partially, and 2: Yes, similar to the Down and Black checklist.^b^ The score for this question was modified as 0, 1, UD to facilitate comparison.

**Table 6:** Methodological quality assessment scores for non-RCT studies.

Author (year)	Reporting								External validity		Internal validity—bias			Internal validity—confounding				Power	Score (%)	Quality
	1	2	3	4	5^a^	6	7	10	11	12	17	18	20	21	22	23	24	27^b^		
Takata et al. (2013) [10]	1	1	0	1	0	1	1	0	UD	UD	1	1	1	UD	UD	0	0	UD	42.1	LQ
Payehdar et al. (2014) [11]	1	1	1	1	2	1	1	1	UD	UD	1	1	1	UD	1	0	0	UD	68.4	HQ
Jung et al. (2022) [12]	1	1	1	1	0	1	1	1	UD	UD	1	1	1	UD	UD	0	0	UD	52.6	HQ

1 = Yes; 2 = No; UD = Unable to Determine; SD: Standard Deviation; HQ: High Quality (Score ≥ 50%); LQ: Low Quality (Score < 50%).

Q1: Clear aim, Q2: Clarity of reporting outcomes, Q3: Clarity of patients’ characteristics, Q4: Describing interventions, Q5: Explaining principal confounders, Q6: Description of main findings, Q7: Estimation and report of random variability, Q10: Reporting actual probability values, Q11: Asked participants well represent the whole population, Q12: The prepared participants well represent the whole recruited participants, Q17: Same time of follow-up, Q18: Appropriate statistical tests, Q20: Accuracy of outcome measures, Q21: Recruiting cases and controls from same population, Q22: Recruiting cases and controls over the same time interval, Q23: Randomized participants to group, Q24: Concealed the intervention from participants and staff, Q27: Sufficient statistical power.

^a^ The score for this question is 0: No, 1: Partially, and 2: Yes, similar to the Down and Black checklist.^b^ The score for this question was modified as 0, 1, UD to facilitate comparison.

**Fig 3:**
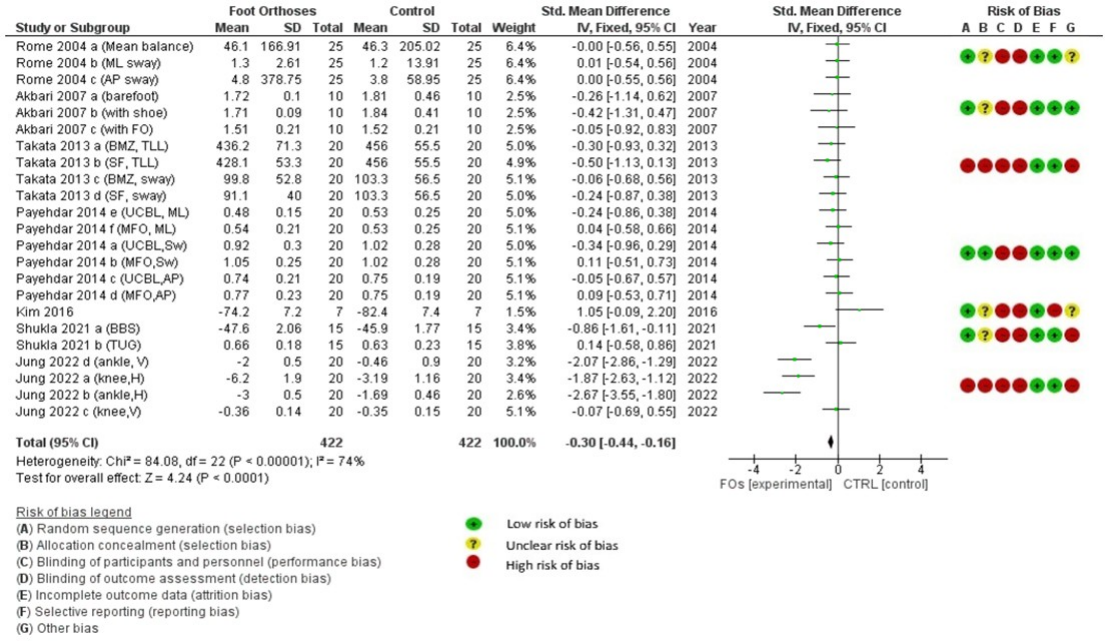
Forest plot of the effect of FOs on balance and risk of bias assessment for all included studies. Note*: Rome 2004 [13] a: Mean balance outcome, Rome 2004 [13] b: Medial-lateral sway outcome, Rome 2004 [13] c: Antero-posterior sway outcome, Akbari 2007 [14] a: Testing in barefoot, Akbari 2007 [14] b: Testing with shoe, Akbari 2007 [14] c: Testing with FO, Takata 2013 [10] a: Total locus length outcome for BMZ, Takata 2013 [10] b: Total locus length outcome for Superfeet, Takata 2013 [10] c: Area of body sway outcome for BMZ, Takata 2013 [10] d: Area of body sway outcome for Superfeet, Payehdar 2014 [11] a: Mean total sway outcome for UCBL, Payehdar 2014 [11] b: Total sway outcome for MFO, Payehdar 2014 [11] c: Antero-posterior sway outcome for UCBL, Payehdar 2014 [11] d: Antero-posterior sway outcome for MFO, Payehdar 2014 [11] e: Medial-lateral sway outcome for UCBL, Payehdar 2014 [11] f: Medial-lateral sway outcome for MFO, Shukla 2021 [5] a: Berg balance scale outcome, Shukla 2021 [5] b: Time-up and go outcome, Jung 2022 [12] a: Horizontal displacement outcome for knee, Jung 2022 [12] b: Horizontal displacement outcome for ankle, Jung 2022 [12] c: Vertical displacement outcome for knee, Jung 2022 [12] d: Vertical displacement outcome for ankle.

Fig 3 illustrates the risk of bias. When selection bias was considered, all RCTs reported low risk in random sequence generation, but no allocation concealment [5,13–15]. While two non-RCT studies demonstrated a high-risk selection bias (no randomized generation) [10,12], one non-RCT study stated that a random intervention sequence and allocation concealment were used [11]. All studies demonstrated a high risk of bias for performance and detection bias because they did not blind any participants, assessors, or outcomes [5,10–15].

In attrition and reporting bias, all studies had a low risk of bias because they reported and analyzed all the outcome measurements and results [5,10–14] except for one study that did not report the actual P-value [15]. Thus, there was a high risk of reporting bias. For the other bias domain, the confounding factors that affect balance outcomes, namely shoe conditions and setting environment, were considered. Two studies had a low risk of bias because they reported all confounding factors [11,14]. Two studies showed unclear risk of bias because they reported only shoes conditions [5,13]. Three studies had high risk of bias because they did not report any confounding factors in their studies [10,12,15].

### Study characteristics

The RCTs included 114 participants (51 men and 63 women). The mean patient age was 20.55 years. The sample sizes of the included studies ranged from 14 [15] to 50 [13]. Studies were conducted in Asia [5,14,15] and the United Kingdom [13]. One study recruited only female participants [14] and another included participants aged from 16 years [5]. Flatfoot was differently diagnosed in the included studies. Akbari et al. [14] diagnosed flatfoot using the arch ratio, Kim et al. [15] based their diagnosis on a navicular drop test, and Rome et al. [13] used the foot posture index. Shukla et al. [5] did not report any diagnostic measurements. The intervention period also varied in each study: Akbari et al. [14] used 2 weeks, Kim et al. [15] used 5 weeks, and Rome et al. and Shukla et al. used 4 weeks [5,13]. Three studies used medial archsupport FOs [5,14,15] and another used rearfoot wedging FOs [13] for their interventions. The control group varied in each study, including soft FOs [14], short-foot exercises [15], no FOs [13], and University of California Berkeley Laboratory (UCBL) FOs [5]. Two studies used laboratory testing for static balance (Biodex System and Balance Performance Monitor) [13,14], while the other two used clinical testing for dynamic balance (Berg Balance Scale, Timed Up and Go test, and Y-balance test) [5,15]. Only one study described the setting [5].

The non-RCTs included 60 participants (32 men and 28 women). The mean patient age was 22.30 years. The sample size of each of the included studies was 20. All studies were conducted in Asia [10–12]. All studies recruited both sexes. Flatfoot was differently diagnosed in the included studies. Takata et al. [10] diagnosed flatfoot by using the bony arch ratio, Payehdar et al. [11] based their diagnosis on the foot posture index, and Jung et al. [12] used the navicular drop test. All the studies used an experimental cross-sectional design. Different types of FOs were used: Takata used cuboid-supported FOs (BMZ) and navicular-supported FOs (Superfeet) [10], Payehdar used UCBLs and modified foot orthoses (MFOs) [11], and Jung used transverse-arch FOs [13]. All three studies were before-and-after design intervention studies. Takata used three conditions: before using FOs (barefoot), BMZ FOs, and Superfeet FOs [10]. Payehdar investigated three conditions: before using FOs (shoes only), while using UCBLs with shoes, and while using MFOs with shoes [11]. Jung used two conditions: before using FOs (barefoot) and when using transverse-arch FOs [12]. All studies used laboratory testing for static balance measurements [10,11,12], including the Zebris system for body sway [10], the Biodex system [11], and two-dimensional video analysis [12]. Only one study described the setting [11].

### Effect of FOs on balance

The meta-analysis incorporated both RCT and non-RCT studies and revealed a significantly favorable outcome for FOs compared to the control group, albeit with high heterogeneity (I² > 75%; Fig 3). Five studies were of high quality [11–15], and two were of low quality [5,10]. Table 7 summarizes the results of the meta-analysis.

**Table 7:** Summary of significant results of meta-analysis.

Analysis	Included study, methodological quality	P-value	Effect size (95% CI)
FOs on balance in all studies	Rome et al. (2004) [13], HQ Akbari et al. (2007) [14], HQ Takata et al. (2013) [10], LQ Payehdar et al. (2014) [11], HQ Kim et al. (2016) [15], HQ Shukla et al. (2021) [5], LQ Jung et al. (2022) [12], HQ	<0.001	SMD -0.03 (-0.04, -0.16)
FOs on balance in non-RCTs	Takata et al. (2013) [10], LQ Payehdar et al. (2014) [11], HQ Jung et al. (2022) [12], HQ	<0.001	SMD -0.43 (-0.60, -0.25)
FOs on balance in high-quality non-RCTs	Payehdar et al. (2014) [11], HQ Jung et al. (2022) [12], HQ	<0.001	SMD -0.49 (-0.70, -0.29)
Transverse-arch insoles on balance	Jung et al. (2022) [12], HQ	<0.001	SMD -1.42 (-1.79, -1.05)

FOs: Foot Orthoses; RCT: Randomized control trial study; non-RCT: Non-randomized control trial study; HQ: High Quality; MQ: Moderate Quality; CI: Confidence Interval.

Effect size is standardized mean difference (SMD). A positive value shows “favor to control” and a negative value shows “favor to foot orthoses” for that parameter during wearing orthoses compared to controls.

**Fig 4:**
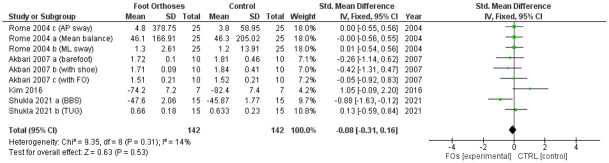
Forest plot of the effect of FOs on balance for RCT studies. Note*: Rome 2004 [13] a: Mean balance outcome, Rome 2004 [13] b: Medial-lateral sway outcome, Rome 2004 [13] c: Antero-posterior sway outcome, Akbari 2007 [14] a: Testing in barefoot, Akbari 2007 [14] b: Testing with shoe, Akbari 2007 [14] c: Testing with FO, Shukla 2021 [5] a: Berg balance scale outcome, Shukla 2021 [5] b: Time-up and go outcome.

The analysis of the RCTs showed mixed results. Three studies that used a medial arch–posting FO [5,14,15] showed a significant improvement in balance before and after using FOs, but they failed when compared with the controls: short-foot exercises [15], soft medial-posting FOs [14], and UCBLs [5]. Another study that used rigid rearfoot posts showed a significant improvement in medial-lateral sway after 4 weeks of using FOs, which was significantly different when compared to the control (no FOs) [13]. The meta-analysis indicated no overall effect of FOs on balance in individuals with flatfoot and showed low heterogeneity (I² < 50%; Fig 4).

**Fig 5:**
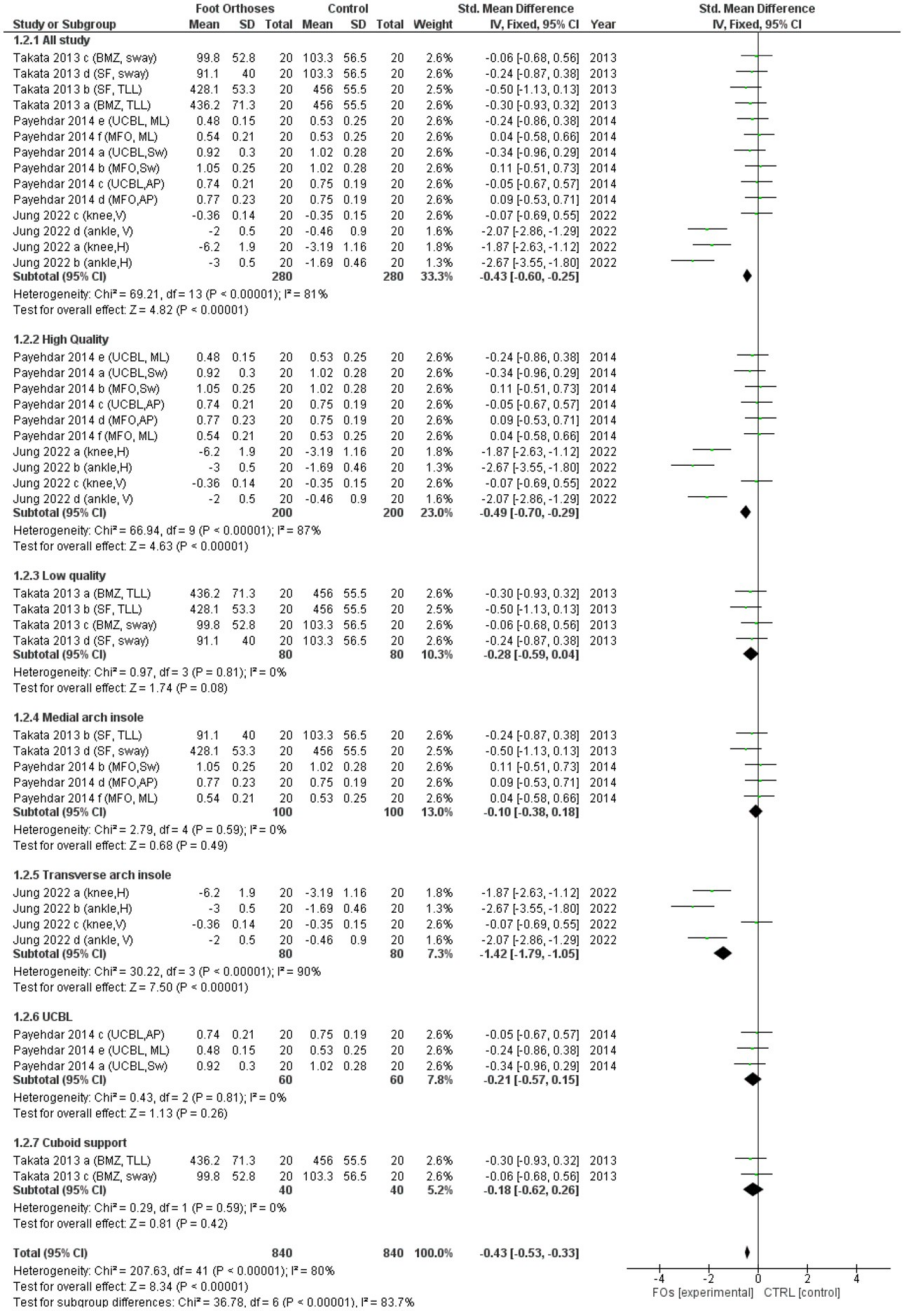
Forest plot of the effect of FOs on balance for non-RCT studies and subgroups of non-RCT. Note*: Takata 2013 [10] a: Total locus length outcome for BMZ, Takata 2013 [10] b: Total locus length outcome for Superfeet, Takata 2013 [10] c: Area of body sway outcome for BMZ, Takata 2013 [10] d: Area of body sway outcome for Superfeet, Payehdar 2014 [11] a: Mean total sway outcome for UCBL, Payehdar 2014 [11] b: Total sway outcome for MFO, Payehdar 2014 [11] c: Antero-posterior sway outcome for UCBL, Payehdar 2014 [11] d: Antero-posterior sway outcome for MFO, Payehdar 2014 [11] e: Medial-lateral sway outcome for UCBL, Payehdar 2014 [11] f: Medial-lateral sway outcome for MFO, Jung 2022 [12] a: Horizontal displacement outcome for knee, Jung 2022 [12] b: Horizontal displacement outcome for ankle, Jung 2022 [12] c: Vertical displacement outcome for knee, Jung 2022 [12] d: Vertical displacement outcome for ankle.

The non-RCT studies, which were all cross-sectional in design, generally indicated an immediate effect of FOs on balance [10–12]. The first study used a medial arch and cuboid insole [10], the second study used UCBLs and MFOs [11] and the third used transverse-arch insoles [12]. Two high-quality studies using medial-posting insoles showed no effect of FOs on balance in flatfoot [11]. In contrast, another low-quality study found an improvement in balance immediately after using FOs [12]. However, a high-quality study using transverse-arch insoles showed a significant improvement in balance after using FOs compared to before using [10]. The meta-analysis revealed that the non-RCT studies significantly demonstrated the effectiveness of using FOs in improving balance compared to the control group (no-FO condition), albeit with high heterogeneity (I² > 75%). Subgroup analysis showed that the high-quality studies significantly favored the use of FOs, again noting high heterogeneity (I² > 75%). In contrast, the low-quality studies did not show any significant effects. Furthermore, when analyzed based on the type of FOs, both groups—those utilizing medial-arch insoles and those using transverse-arch insoles—significantly favored the use of FOs, despite high heterogeneity (I² > 75%), as illustrated in Fig 5.

**Fig 6:**
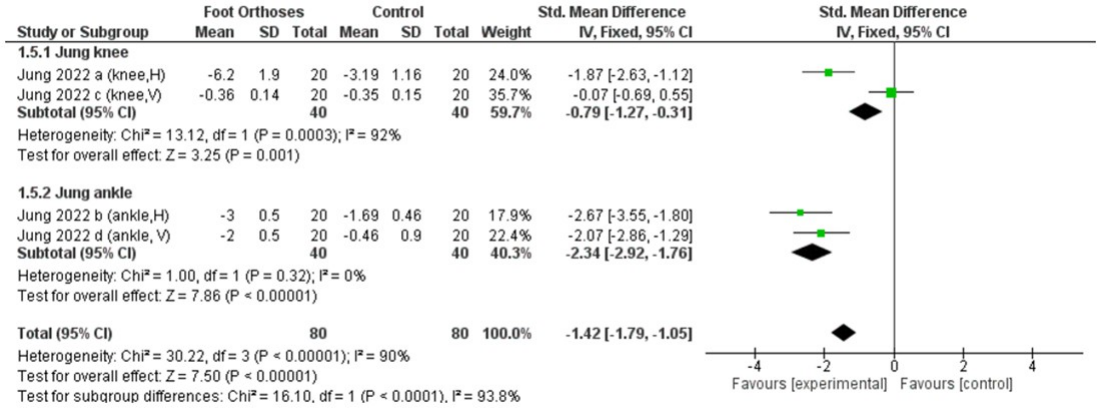
Forest plot of the subgroups of results from the effect of FOs on transverse arch insole study (Jung et al. [12]). Note*: Jung 2022 [12] a: Horizontal displacement outcome for knee, Jung 2022 [12] b: Horizontal displacement outcome for ankle, Jung 2022 [12] c: Vertical displacement outcome for knee, Jung 2022 [12] d: Vertical displacement outcome for ankle.

In the subgroup analysis of the non-RCTs, high heterogeneity (I² > 75%) was found in the high-quality and transverse-arch insole groups. A transverse-arch insole test was also performed in the high-quality group. Therefore, the outcomes of the transverse-arch insole study were presented as sub-groups. Both knee and ankle displacement outcomes showed a significant effect; however, knee displacement showed high heterogeneity (I² > 75%), whereas ankle displacement showed low heterogeneity (I² < 50%), as illustrated in Fig 6.

## Discussion

This meta-analysis is the first to explore the effect of FOs on balance in individuals with flatfoot. The systematic review encompassed seven experimental trials with a quality spectrum ranging from low to high, comprising four RCTs and three non-randomized studies [5,10–15]. The primary outcome of the review was immediate improvement in balance when medial or transverse-arch insoles were used. However, this beneficial effect did not persist when FOs were employed for 2–5 weeks in comparison to a control group. This transient enhancement of balance is consistent with previous research suggesting that FOs may temporarily realign the lower extremities [30] and augment muscle function [31], thereby facilitating better coordination and postural control. The absence of sustained effects raises questions regarding the long-term efficacy of FOs in balance enhancement and suggests that the mechanisms for immediate improvement may not translate into long-term functional adaptations.

### Methodological considerations

Seven studies with both RCT and non-RCT designs were included in the methodological assessment. The quality of the studies varied, ranging from low to high [5,10–15]. Methodological inconsistencies, particularly in the RCTs, such as inadequate descriptions of treatment procedures and incomplete reporting of potential confounders, challenge the internal validity and reliability of these findings. Specifically, 25% of the RCTs [5] failed to detail the intervention protocols, a critical element for the replicability and interpretation of results. Confounders were either fully or partially addressed in 75% of RCTs [13–15], with complete omission in one study [14]. Such an oversight could potentially skew the perceived effectiveness of the interventions.

Concerns extend to the presentation of statistical measures. One RCT [15] neglected to report the P-values and psychometric properties of the outcomes, which are key factors for evaluating the strength of evidence. Furthermore, half of the RCTs provided no insight into the selection process for their source populations, limiting the findings’ applicability across different populations [5,13]. The timing of participant recruitment, a detail omitted in 75% of the RCTs [5,13,14], is also crucial for consistency and has not been uniformly reported.

The non-RCTs exhibited similar reporting gaps, with 33% [10] not disclosing the source population characteristics and only one [11] fully reporting both the principal confounders and participant recruitment timing, which are vital for limiting and understanding the bias. Although P-values were reported in 66% [11,12] of the non-RCT studies, the absence of such details in the remaining studies could have influenced the credibility of the results.

The risk of bias assessment revealed a high risk of bias for several aspects. First, all of the RCT studies did not report allocation concealment [5,13–15]. Second, none of the studies were blinded to any measurements in their studies [5,10–15]. Third, some studies avoided reporting all the confounding factors that could affect the results [5,10,12,13,15]. The results of this meta-analysis could be biased, due to the biased results in the included trials.

When publication bias was considered, the funnel plot was asymmetric (Fig 2). This could be attributed to the fact that the effects of interventions reported in smaller studies differ from those calculated in larger studies [32]. Differences in methodological quality are a key potential source of funnel plot asymmetry, although true heterogeneity in intervention effects can also contribute [23]. It is feasible that the asymmetrical funnel plot was the result of publication bias.

Future studies should address these methodological deficiencies to maintain the research integrity. Detailed reporting of intervention protocols, confounder management, and statistical outcomes is crucial for robust and reproducible research. Such transparency in methodological reporting enhances confidence in the conclusions drawn and strengthens the overall evidence.

### Study characteristics

The meta-analysis revealed a significant improvement in balance with the use of FOs in individuals with flatfoot (P < 0.001, I^2^ = 74%), encompassing both RCT and non-RCT studies. However, closer examination revealed a nuanced picture; although non-RCT studies uniformly indicated improvement (P < 0.001, I^2^ = 81%), RCTs did not demonstrate a significant change (P = 0.54, I^2^ = 15%). This discrepancy may be attributed to the different outcome measures employed across the RCTs, ranging from the Biodex system to the Y-balance test, despite the use of similar FO types. Such variability, along with differing diagnostic criteria for flatfoot, may have contributed to the mixed results, suggesting that the choice of balance metrics can heavily influence study outcomes.

Furthermore, the duration of the FO intervention varied, with most RCTs applying a 4–5 week period [5,13,15], except for one study that assessed balance over 2 weeks [14]. This variation may account for the observed differences in efficacy. In the non-RCTs, the advanced instruments used to measure postural sway, such as the Zebris system, demonstrated significant effects on balance. Interestingly, the quality of these studies was a determining factor, with high-quality studies reporting more pronounced improvements, underscoring the importance of methodological robustness.

Particularly notable is the finding that among the different types of insoles evaluated in the non-RCT studies, only transverse insoles significantly enhanced static balance (P < 0.001, I^2^ = 90%) [12]. This suggests the specific efficacy of transverse insoles, warranting further investigation. Only two RCTs addressed the dynamic balance, with divergent indications of quality and outcomes.

These findings collectively highlight the complexity of assessing the impact of FOs on balance and underscore the need for the standardization of outcome measures and diagnostic criteria to facilitate clearer interpretation and applicability of results in clinical practice.

### Effects of FOs on balance

The meta-analysis assessed the efficacy of FOs on balance across studies, with methodological quality ranging from low to high. Meta-analysis of all studies favored FOs with high heterogeneity (I^2^ >75%). This indicates variance in methodological approaches among the included studies, encompassing both RCT and non-RCT designs.

The RCT meta-analysis revealed no significant improvement in balance when compared to the control group, with low heterogeneity (I^2^ < 50%). This finding suggests methodological consistency across these studies, yet an absence of efficacy of FOs in enhancing balance in flatfoot over the evaluation period of 2–5 weeks. These studies sought to determine whether FOs could augment proprioceptive acuity based on evidence suggesting that orthotic pressure enhances joint proprioception and, subsequently, balance [33,34]. However, the literature on proprioceptive training indicates that effective proprioceptive improvements typically require regimens that extend beyond 5 weeks [35].

Conversely, the non-RCT studies, often employing a cross-sectional design, demonstrated an improvement in balance with the use of FOs, as indicated by the high heterogeneity (I^2^ > 75%). This supports the theory that balance, an intricate motor skill, is sustained by a confluence of cognitive, motor, and sensory systems [36,37], which are potentially modulated by the structural and functional support provided by the FOs [38]. Specifically, transverse-arch insoles are notable for their positive effect on static balance, possibly because they enhance foot stiffness and energy storage, contributing to foot alignment and support [12]. However, significant heterogeneity across these non-RCT studies, characterized by diverse FO types and outcome measures, necessitates a cautious interpretation of these findings. Furthermore, the subgroup analysis of transverse-arch insoles showed low heterogeneity (I^2^ < 50%) in ankle displacement outcomes. This indicates that this type of insole can increase ankle stability. The ankle joint has minimal movement, allowing the body to act as a single-segment inverted pendulum to promote balance [39]. When considering knee displacement outcomes, the results revealed a significant effect but with high heterogeneity (I^2^ > 75%). There is evidence that poor ankle stability could affect balance and neuromuscular control of the proximal joints (i.e., the knee and hip) [40]. Thus, improving ankle joint stability due to transverse arch support may have a significant influence on knee joint stability, although caution should be exercised when interpreting this result.

### Clinical implications

The findings of this systematic review and meta-analysis suggest that FOs provide beneficial effects. In this study, among several types of insoles, i.e. medial arch insole, UCBL, cuboid support, and transverse-arch insole, only transverse-arch insoles improved static balance immediately after use. Clinicians can apply the findings of this study to clinical settings but with caution, because the study revealed some internal validity and publication bias. In addition, the long-term effects of FOs require further investigation.

The benefit of using an inserted arch for controlling flatfoot during weight-bearing functional activities is crucial, as a normal arch of the foot assists in providing an upright posture and weight bearing as well as absorbing the shock generated during locomotion [41,42]. With an inserted arch, proper alignment of the trunk and lower extremities can be maintained [43,44], and with subsequent improvement in balance [5,10,12–15]. This conceivable advantage may be specifically useful for people with severe flatfoot or the elderly who easily lose balance and are at a high risk of falling. Although previous evidence has demonstrated that exercise is the best method for improving balance and preventing falls [45,46], individuals with severe flatfoot or of older age may need a method that produces rapid changes in the arch of their feet; therefore, insoles can be one of the treatment options.

### Limitations and strengths

This study presents a comprehensive meta-analysis of the strengths and limitations of the effects of FO on balance in individuals with flatfoot. Methodologically, it included a systematic search of full English texts, providing a detailed overview of the available evidence, while acknowledging the possibility of missing relevant studies in other languages. The inclusion of both RCTs and non-RCTs enriched the diversity of the data, offering a wide lens through which to assess the efficacy of FOs, although it also introduced varying levels of evidence that could influence the overall findings. This systematic review and meta-analysis included studies with publication bias and a high risk of bias in some aspects of internal validity (allocation concealment, blinding, and reporting of confounding factors). Caution should be exercised when interpreting the results. This study contributes significantly to a field where systematic reviews and meta-analyses are scarce, thereby setting a foundation for future inquiry and bolstering the understanding of the roles of FOs in clinical practice.

The strength of this study lies in its systematic approach and broad inclusion criteria, which highlight the current evidence and suggest directions for future research. Despite the potential for publication bias due to language restrictions, the findings of this study are pertinent to the existing literature and provide a valuable reference point for subsequent studies. Furthermore, the use of UCBL orthoses in different capacities across study designs prompts a critical analysis of the data and underscores the necessity for consistent control interventions in research. This meta-analysis not only maps out the existing terrain, but also elucidates the methodological nuances critical for advancing research on the therapeutic use of FOs for balance improvement in flatfoot.

## Conclusion

FOs with transverse arch support have been shown to produce immediate improvements in static balance in individuals with flatfoot, a finding supported by high-quality evidence. However, this effect appeared to be transient because the continued use of FOs for 2–5 weeks did not sustain balance enhancement. This meta-analysis also highlighted significant methodological variability across studies, including differences in participant populations, orthotic designs, outcome measures, and diagnostic criteria. These disparities underscore the need for greater uniformity in future studies. Standardization in the recruitment of participants and implementation of FO interventions is crucial to ensure the replicability and reliability of the findings. Although the immediate benefits of FOs are evident, more rigorous and harmonized research is necessary to determine their long-term efficacy and optimal use in patients with flatfoot.

## Supporting information

S1 Checklist PRISMA 2009 checklist. (PDF)
